# P02.99. Acupuncture combined with an antidepressant has a better effect on major depression: a multi-center, randomized, controlled clinical trial

**DOI:** 10.1186/1472-6882-12-S1-P155

**Published:** 2012-06-12

**Authors:** T Guo, Y Wang, L Sun, W Zhang, W Ma

**Affiliations:** 1 Beijing University of Traditional Chinese Medicine, Beijing, China

## Purpose

This study investigated whether the combination of acupuncture and the antidepressant medicine paroxetine has a better curative effect than paroxetine only for major depression (MD) in a multi-center, randomized, controlled clinical trial.

## Methods

72 inpatients and outpatients with MD, diagnosed by the ICD-10, were randomly divided into three groups with three different treatments: combination of manual-acupuncture and paroxetine (23 cases); combination of electro-acupuncture and paroxetine (32 cases); and paroxetine only (17 cases) for 6 weeks. Two statistical analyzing methods, intention to treat (ITT) and per protocol (PP), were applied to assess the main curative indexes, including Hamilton Depression Scale (HAMD), Self-rating depression scale (SDS), and Rating Scale for Side Effects (SERS) scores.

## Results

Numbers of patients dropping out during the treatment were 3, 5, and 1 in the three groups. Patients in the two acupuncture groups got a more remarkable reduction in HAMD and SDS scores compared with those in the medicine only group, especially in the somatic factor scores. No significant differences were found between the two acupuncture groups (p>0.05). SERS scores in the 2^nd^, 4^th^ and 6^th^ week showed significant differences among the three groups (p<0.05).

## Conclusion

The findings indicate that the combination of acupuncture and antidepressant is superior to the antidepressant treatment only in term of improving patients’ depressive symptoms, especially somatic symptoms. Acupuncture combined with antidepressant medicine can substantially improve patients’ subjective feelings for quality of life. Acupuncture plays an important role in reducing the side effects caused by the antidepressant medicine and enhancing its curative effects.

